# Cellular Fibroma of the Ovary with Multiloculated Macroscopic Characteristics: Case Report

**DOI:** 10.1155/2012/283948

**Published:** 2012-04-11

**Authors:** Sheila Jorge Adad, Valeria Lima Laterza, Carlos David Teixeira dos Santos, Antonio Alexandre Lisboa Ladeia, Joao Carlos Saldanha, Cleber Sergio da Silva, Luis Ronan Marquez Ferreira e Souza, Eddie Fernando Candido Murta

**Affiliations:** ^1^Discipline of Special Pathology, Universidade Federal do Triangulo Mineiro (UFTM), Avenida Getulio Guarita 130, 38045-440 Uberaba, MG, Brazil; ^2^Discipline of Gynecology and Obstetrics, Universidade Federal do Triangulo Mineiro (UFTM), Avenida Getulio Guarita 130, 38045-440 Uberaba, MG, Brazil; ^3^Discipline of Radiology and Diagnostic Imaging, Universidade Federal do Triangulo Mineiro (UFTM), Avenida Getulio Guarita 130, 38045-440 Uberaba, MG, Brazil

## Abstract

Ovarian fibroma is the commonest benign tumor of the ovarian stroma. The cellular subtype accounts for around 10% of ovarian fibromatous tumors. The cellular fibroma is a tumor of uncertain malignant potential that may recur or be associated with peritoneal implants. Usually these are solid tumors, sometimes with small areas of cystic degeneration. This case is reported to highlight an unusual feature for an ovarian fibroma: the tumor was predominantly cystic with a small solid part; the multiple cavities contents consisted of viscous liquid that solidified under room temperature. The multiloculated cysts, the mucinous contents, and the solid areas simulated a borderline mucinous ovarian tumor on both CT scan and gross pathologic examination.

## 1. Introduction

Ovarian fibroma is the commonest benign tumor of the ovarian stroma (4% of all ovarian neoplasms), and it can occur at any age [1]. The cellular subtype, approximately 10% of ovarian fibromatous tumors, exhibit hypercellularity, increased mitotic activity, and mild-to-moderate nuclear atypia [2]. The cellular fibroma is a tumor of uncertain malignant potential that may recur or be associated with peritoneal implants. The degree of mitotic activity is the main parameter for differentiating cellular fibroma from fibrosarcoma [2]. Fibrosarcomas usually exhibit moderate-to-severe nuclear atypia and mitotic counts of 4 or more per 10 high-power fields (HPFs). Macroscopically, the cellular fibroma has a whitish appearance resembling uterine leiomyoma, a generally solid consistency and, sometimes, small areas of cystic degeneration and stromal edema. Clinically, it is asymptomatic and may typically be detected during routine gynecological examinations. Its behavior is usually benign, but the completeness of excision and presence or absence capsule rupture are important prognostic parameters [3]. 

## 2. Clinic Case

**Figure 1:**
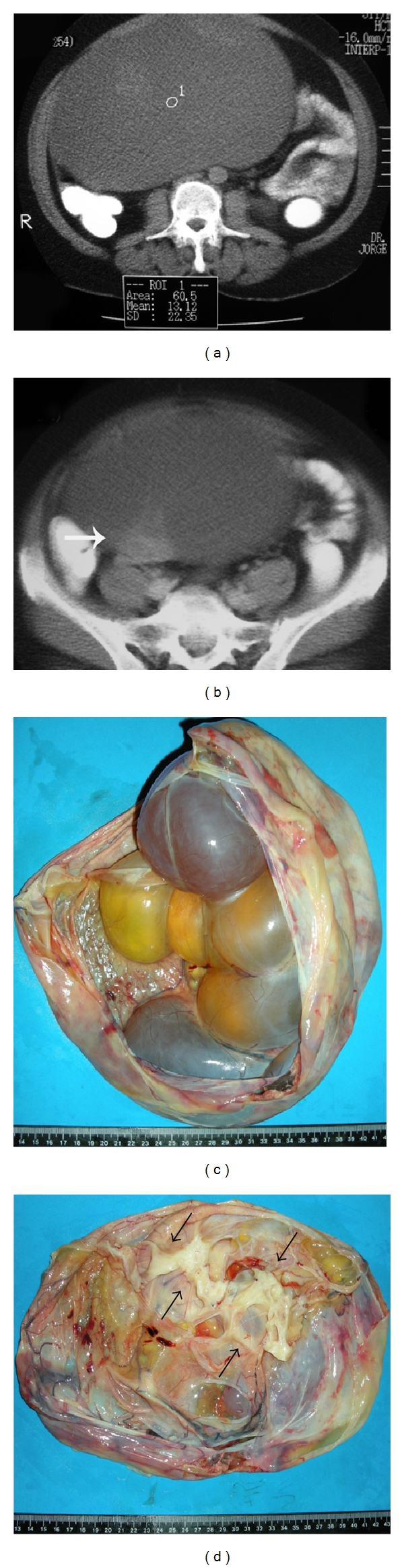
(a) Not contrasted helical computed tomography (CT) of the abdomen; large cyst (water ROI mean 0–20 HU; this cyst 13.12 HU) with well defined limits and finely heterogeneous appearance; (b) Contrast enhanced helical CT of the abdomen; arrow indicates a contrast enhanced solid area in the cyst wall, suggesting malignancy; (c) Partially opened surgical specimen showing the multiloculated cystic appearance, with mucinous content and thin wall; (d) Solid, whitish, and elastic area seen at depth, which corresponded microscopically to the regions with more cellularity.

A 53-year-old woman (G4N4A0) suffered right-side abdominal pain for eight months, associated with abdominal distension, increased abdominal size, nausea and vomiting. Ultrasonography (6 months before surgery): cystic image with smooth surfaced wall and coarse septa in the center; hypovascularization to Doppler study, measuring 19.9 × 12.9 × 19.2 cm; volume: 2562 cm³; located in the middle region, occupying the upper and lower abdomen. Computed tomography (CT)—5 months before surgery: a complex large-volume lesion extending from the pelvis to the upper abdomen (20 × 20 × 15 cm; volume: 3000 mL), without signs of ureteral compression, ascites or enlarged lymph nodes (Figures 1(a) and 1(b)). Tumor markers CA 125, CA 15.3, CA 19.9, CEA, *α*-fetoprotein, and *β*-HCG were negative. Exploratory laparotomy revealed a right-side adnexal cystic and a moderate quantity of ascitic fluid. The uterine annexal cystic tumor was sent to Department of Pathology to be analyzed by frozen biopsy. The lesion was considered consistent with cystic tumor of borderline malignancy, mucinous probably, or association between two tumors. The patient then underwent expanded total abdominal hysterectomy. The ascitic fluid, right and left parietocolic gutter, right and left pelvic lymph nodes, infracolic omentum, hepatic capsule, uterus, and contralateral ovary were free from neoplasia. 

## 3. Pathologic Study

Gross pathologic findings: the ovarian tumor measured 27 × 27 × 15 cm and weighed 6630 g, with smooth grayish external surface and absence of macroscopic rupture. Upon opening the cystic tumor, a multiloculated appearance with cavities was observed (Figure 1(c)). The tumor was covered with a thin wall of 0.1 cm in thickness that was generally smooth except in one area measuring 5 × 3 × 2 cm, that was solid, whitish and with fibroelastic feature (Figure 1(d)). The cavities were filled with a clear viscous fluid when fresh, but it gradually turned gelatinous material under room temperature, suggesting mucinous tumor. Epithelium was not seen lining the cysts in the frozen sections biopsy. However, the macroscopic features of multiloculated cysts, mucinous contents, and the solid area, which was microscopically hypercellular with mild atypia, led to the diagnosis of cystic tumor of borderline malignancy, mucinous, probably or association between two tumors on frozen biopsy. The remaining material was then fixed in buffered formalin and processed for paraffin, submitted after the microtome, and stained by Hematoxylin-eosin (HE) and immunohistochemical; 30 blocks were made of the neoplasm by sampling, extensively, the solid and cystic areas. 

**Figure 2:**
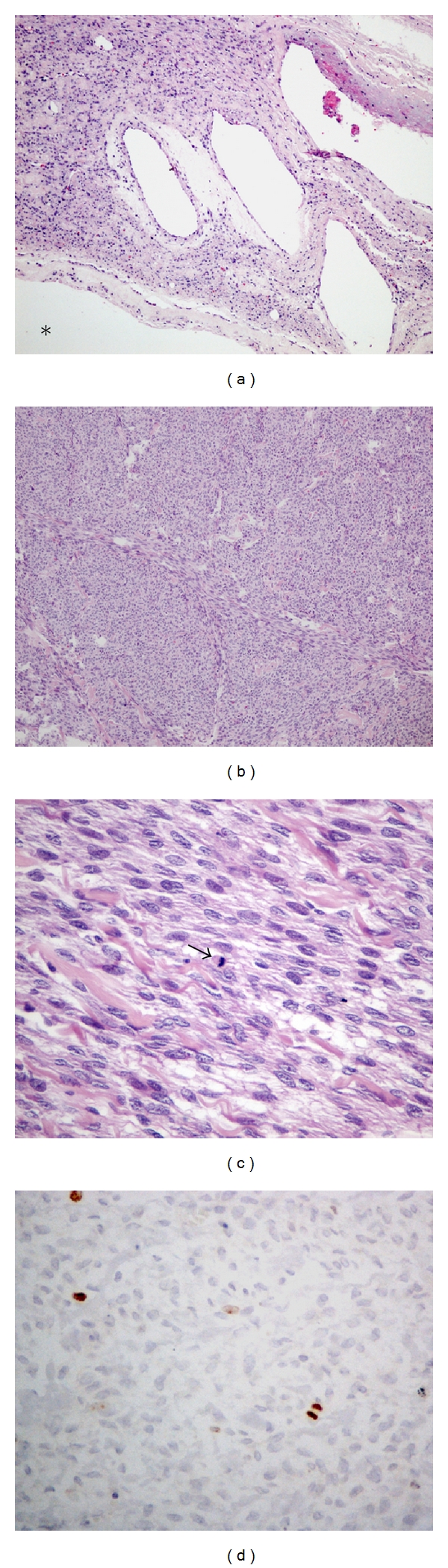
(a) Photomicrograph of the wall of the cystic area (asterisk inside the larger cavity) showing no epithelium lining and other similar, smaller cavities, with fibroma in the middle, with usual cell density (HE—100x); (b, c) and** (**d) Representation of the solid area compatible with cellular fibroma, noting evident high cell density ((b) HE-100x), mild atypia ((c) HE—400x) and mitosis (arrow); (d) MIB-1 immunostaining (400x) showing sparse positive cells (2-3%).

Microscopically, the tumor showed alternating solid and cystic areas. However, there was no epithelium lining the cavities at any location; the cavities were limited by granulation tissue that, in turn, was surrounded by myxoid alteration. Part of the solid areas were slightly hypercellular with nonatypical spindle cells arranged in storiform pattern, compatible with fibroma (Figure 2(a)). Frequently small foci were observed with incipient cystic degeneration. The macroscopically largest solid area was moderately to highly cellular with slight nuclear atypia and scant mitotic figures. Mitotic figures were counted in 100 HPF, divided into 10 sets of 10 fields; no more than 3 mitoses/10 HPF were seen (Figures 2(b) and 2(c)). Immunohistochemical analysis was performed using the polymer technique with the following sera: anti-cytokeratin (clone AE1/AE3, Dako, 1 : 400), anti-EMA (clone E29, Dako 1 : 200), CD34 (clone QBEnd/10; Novocastra, 1 : 600), anti-vimentin (clone VIM3B4, Dako 1 : 1200), antismooth muscle actin (clone 1A4, Dako, 1 : 200), anti-desmin (clone D33, Dako, 1 : 100), and anti-ki67 (clone MIB-1, Dako, 1 : 500). Immunohistochemical analysis revealed that the tumor was negative for AE1AE3, EMA, CD34 and desmin, while focally positive for vimentin and actin 1A4. MIB-1 (Figure 2(d)) was positive in 2-3% tumor cells (based on 1000 tumour cells count). We concluded, after its inclusion in paraffin, that it was a cellular fibroma. 

After surgery patient progressed well and is currently living in good health and without signs of tumor recurrence (follow up: 33 months). 

## 4. Discussion

This case is interesting in several respects. The patient's complaints were gastrointestinal disorders, characterized by colic pain, bloating, nausea, and vomiting that started and had been progressing for about 8 months. The first place she sought medical care was at the clinic of digestive diseases. After voluminous abdominal mass was detected by imaging studies, she was referred for gynecologic evaluation. This fact is not new in relation to ovarian neoplasms. Early ovarian cancer-associated symptoms constitute a constellation of mostly nongynecological complaints, suggesting a visceral disturbance, which do not point immediately to a pelvic origin. Abdominal bloating and pain predominate with recent onset and multiple symptomatic episodes [4]. Gastrointestinal and urinary symptoms and fatigue/malaise may be part of the symptom complex. These symptoms are caused by compression of the stomach and intestine by increased adnexal. In the study of Irving et al. [3], the mean age of 35 patients with benign cellular fibroma was 51 years, roughly the same age as our patient.

Although the patient had ascites, commonly found in women with ovarian fibroma, both serum CA 125 and chest X-ray did not show the presence of pleural effusion. These findings departed the diagnosis of Meigs' syndrome. “This syndrome may be suspected when faced with an important pleural effusion, a very elevated CA-125 serum level, a negative cytologic examination of the ascitic effusion and no peritoneal implant on CT-scan” [5].

 The surgical time was based on the diagnosis of cystic tumor of borderline malignancy by frozen biopsy, patient's age, CT, and intraoperative findings which showed ascites.

 Cellular fibromas are predominantly solid. Cystic areas are usually small and without multiloculation. But “some fibromas undergo proeminent cystic degeneration and may be misconstrued as surface epithelial stromal tumours. However, the cyst (pseudocyst) do not have an epithelial lining.”[6]. We found no reports of cellular fibroma with multicystic mucinous content. We found one report of cellular fibroma entirely multicystic with no solid areas but the authors do not relate the contents [3], and another case predominantly cystic [2], however the authors did not describe whether the tumor was multicystic or not and the content type. In this present case there was a predominance of cystic component; due to solid/cystic feature with multiple cysts containing mucinous material, the tumor simulated a borderline mucinous ovarian tumor on both CT and gross pathological examination. The patient was treated on the basis of this diagnosis; but considering that the final diagnosis was a cellular fibroma, the ovary could have been the only structure removed. However, cellular fibroma is a tumor of uncertain malignant potential. It is capable of locally aggressive growth if incompletely excised [2]. Moreover, metastasis occasionally occurs, indicating that criteria for differentiating between cellular fibroma and fibrossarcoma are imperfect. In the study of Prat and Scully, the microscopic feature that correlated best with prognostic was the amount of mitotic activity, with the degree of nuclear atypia less helpful [2]. In this series, 4 of 11 cases of cellular fibroma with atypia degree 2 in 3 did not have any complication or recurrence. They also emphasize that “completeness of removal of the tumor has been considered in determining the prognosis as well as mitotic count” [2].

Irving et al. [3] studied 75 cases of cellular fibromas of the ovary and suggested the term “mitotic active cellular fibroma (MACF)” to cellular fibromatous neoplasm with bland cytology with ≥4 mitosis/10 HPF. They pointed out that the term fibrosarcoma should be reserved for fibroblastic tumour with moderate or severe atypia and high mitotic rate. They still suggested that aggressiveness could occur in cellular fibromas with adhesions at the time of resection or tumor rupture during surgery [3]. In the present case, there were no adhesions or rupture of the tumor. The patient is alive 33 months after surgery, without recurrence or metastases. The tumor was widely sampled for microscopy (30 blocks), considering its unusual feature. No epithelium was found lining the cavities. There were several areas of cystic degeneration, where the tumor was less cellular. In hypercellular areas mild atypia and scattered mitoses were found having differential diagnosis with fibrosarcoma. Nevertheless, fibrosarcoma usually exhibit a combination of moderate-to-severe atypia, high mitotic rate and an aggressive clinical course [3]. But recently a case of well-differentiated ovarian fibrosarcoma has been described, with low-grade cytological atypia and 1-2 mitosis/10 HPF developing liver metastasis one year later [7]. Those authors suggested that in addition to the macroscopic aspect, high rates for Ki-67 (MIB-1) could contribute to the diagnosis of malignancy, despite having a low visual mitotic rate.

 Other authors also considered MIB-1 to be a useful additional parameter for distinguishing between ovarian cellular fibroma and fibrosarcoma [8]. Huang et al. [9], in a retrospective study, also concluded that mitotic activity, and MIB-1-positive cells were identified as important factors in the diagnosis of ovarian fibrosarcoma. Usually, cellular fibromas showed less than 3% of MIB-1-positive cells. Delay in fixation changes mitotic rate; “decrease in counts is largely due to their reduced identifiability and only partly attributable to a completion of the cell cycle.” However, the flow cytometric data did not change substantially [10]. Perhaps a reduction of identifiable mitoses occurred in a recently reported case [7], due to extensive ischemic changes, while the positivity rate for MIB-1 that was surprisingly high (60%). However, in the case described here, both the rate of mitosis (maximum 3 mitoses/10 HPF) and MIB-1 (2-3%), as well as alternating areas of low and high cellularity, no adherence or rupture and no aggressive clinical course findings are consistent with a cellular fibroma diagnosis.

In conclusion, ovarian cellular fibromas are usually solid and when cysts are present they are often scant and small. In the present case, the multiloculated cysts, the mucinous contents and the solid areas simulated a borderline mucinous ovarian tumor on both CT and gross pathological examination.
